# The Natural History of Femoroacetabular Impingement

**DOI:** 10.3389/fsurg.2015.00058

**Published:** 2015-11-16

**Authors:** Benjamin D. Kuhns, Alexander E. Weber, David M. Levy, Thomas H. Wuerz

**Affiliations:** ^1^Department of Orthopedic Surgery, Division of Sports Medicine, Hip Preservation Center, Rush University Medical Center, Chicago, IL, USA; ^2^Division of Sports Medicine, Center for Hip Preservation, New England Baptist Hospital, Boston, MA, USA

**Keywords:** femoroacetabular impingement, hip osteoarthritis, hip preservation surgery, FAI etiology, hip arthroscopy

## Abstract

Femoroacetabular impingement (FAI) is a clinical syndrome resulting from abnormal hip joint morphology and is a common cause of hip pain in young adults. FAI has been posited as a precursor to hip osteoarthritis (OA); however, conflicting evidence exists and the true natural history of the disease is unclear. The purpose of this article is to review the current understanding of how FAI damages the hip joint by highlighting its pathomechanics and etiology. We then review the current evidence relating FAI to OA. Lastly, we will discuss the potential of hip preservation surgery to alter the natural history of FAI, reduce the risk of developing OA and the need for future arthroplasty.

## Introduction

The management of femoroacetabular impingement (FAI) is a rapidly developing field in orthopedics. Described by Ganz in 2003, FAI is a pathologic condition resulting from abnormal acetabular and femoral head/neck morphology that has been implicated as a precursor to secondary osteoarthritis (OA) (1–3). However, the relationship between FAI and OA is not straightforward as there exists a large asymptomatic population and without radiographic signs of OA that possesses the morphologic characteristics of FAI (4). While initially managed conservatively, symptomatic FAI is often treated surgically with the goals of relieving pain, increasing range of motion, and preventing or delaying OA and the potential need for total hip arthroplasty (THA). As FAI is increasingly diagnosed in a younger and more active population, the link between high intensity athletic participation during adolescence and the onset of FAI is under investigation (5). The purpose of this article is to review our current understanding of FAI by focusing on the mechanisms of injury, etiology, treatment strategies, and the debate about its predisposition to OA.

## How Does FAI Damage the Hip Joint?

**Figure 1:**
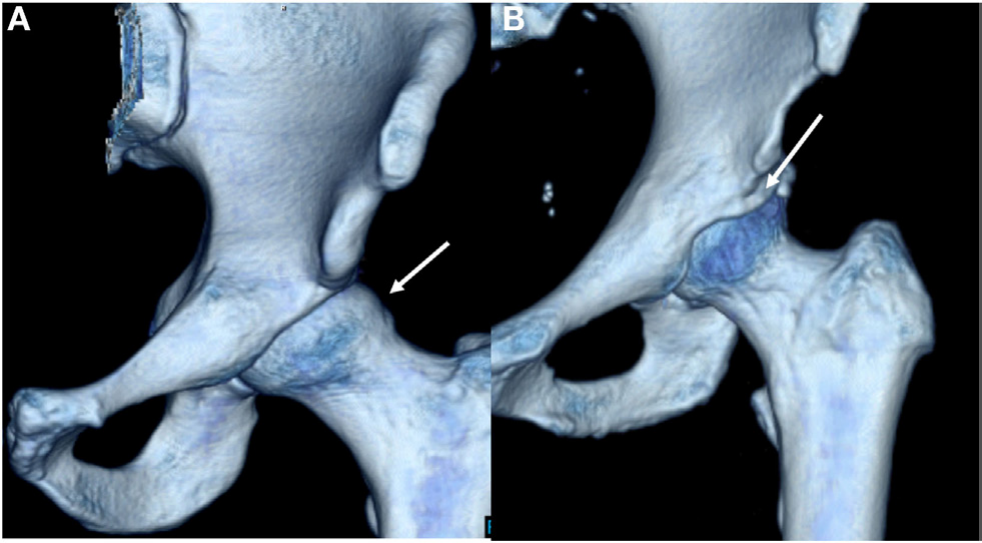
**Three-dimensional CT reconstructions demonstrating cam (A) and pincer (B) deformities**.

Femoroacetabular impingement results from femoral and acetabular incongruity that induces labral, and chondral damage, causing pain and restricting mobility. Cam lesions at the femoral head/neck junction as well as pincer lesions signifying acetabular overcoverage comprise the osseous deformities of FAI (Figure 1A) (1, 6). Termed “mixed” lesions, commonly FAI is a combination of both with varying degrees, but cam and pincer lesions also occur in isolation. One recent systemic review of 1130 hips found mixed impingement in 45% of cases (7–9). While both lesions are seen in FAI, they result in distinct patterns of articular damage which are markedly different (Table 1). Pincer lesions can vary in severity from focal overgrowth of the anterior acetabular rim with acetabular retroversion to the more global deformities seen with coxa profunda or protrusio acetabuli (Figure 1B) (2). The pincer deformity initially damages the labrum when the hip is in flexion, which brings the acetabular overgrowth into apposition with the femoral neck, thereby compressing the anterior labrum (3, 6). With repeated hip flexion, the labrum sustains repetitive microtrauma gradually separating from the acetabular cartilage and eventually failing (2). As the disease progresses, persistent pressure between the posteroinferior acetabulum and the posteromedial aspect of the femoral head initiates acetabular cartilage damage known as the “contrecoup” lesion (10, 11).

**Table 1:** **General characteristics of cam and pincer deformities in FAI**.

	Cam	Pincer
Presentation	Hip/groin pain	Hip/groin pain
Demographic trends	Younger males	Young–middle-aged females
Osseus morphology	Aspherical bump at the femoral head–neck junction, decreased femoral head–neck offset	Focal or global acetabular overcoverage; acetabular retroversion
Injury mechanism	Primarily affects cartilage with repeated flexion. The labrum gets damaged secondarily	Affects labrum primarily; with damage patterns located on the peripheral acetabulum. Associated with contrecoup lesions
Radiographic predictors	Pistol grip deformity; alpha angle >50°	LCEA > 39°; ACEA > 39°, posterior wall sign

Cam deformities present with femoral head asphericity, seen as a flattening of the anterior contour of the head/neck junction or an osseous bump producing a decreased femoral head–neck offset. The bump is often located in the anterolateral or anterosuperior region of the head–neck junction and can be identified as a “pistol grip” deformity on AP and modified Dunn radiographs (3, 11). Similar to the pincer lesion, cam impingement is most symptomatic with the involved leg in flexion (12). However, unlike pincer lesions, the mechanism of impingement is through shear stress generated as the femoral lesion rotates through the anterosuperior acetabulum (11). From a clinical standpoint, the mixed variant of FAI can present with variable degrees of both injury patterns depending on the predominance of the existing lesions (3).

While osseous deformities underlie FAI, symptoms usually result following labral and chondral injury secondary to the impingement itself. In a recent study, Clohisy et al. found that 93% of patients undergoing surgery for FAI had associated labral injury, and 83% had associated cartilage damage (9). Labral and cartilage injuries occur by different mechanisms for cam and pincer lesions. Patients with pincer impingement present primarily with labral damage, consistent with the pathomechanics of acetabular overcoverage (12). Cartilage lesions in patients with pincer deformities are distinct, with acetabular chondral injury occupying a narrow circumferential band that is less severe than those with cam deformities (7, 12). Additionally, repeated microtrauma to the labrum in pincer abnormalities initiates bone growth at the acetabular rim and promotes eventual labral ossification.

For cam deformities, as the eccentric aspect of the lesion passes through the anterosuperior acetabulum during flexion, the transition zone between the labrum and acetabular cartilage is subjected to compressive and shear stresses (11). This causes the labrum to translate away from the joint while the cartilage is pushed in the opposite direction, preserving the labrum until later in the disease process (7, 12). Consequently, cam lesions initially damage the acetabular cartilage, causing delamination of the cartilage from the labrum, compared to pincer lesions which affect the labrum primarily (3, 11). As the mixed variant of FAI is common, patients frequently present with evidence of both chondral and labral damage resulting from cam and pincer deformities, respectively (3). Notably, poor preoperative cartilage status in symptomatic FAI patients is associated with delayed time till surgery and is a harbinger of potentially worse outcomes (13–15). Overall, the bony lesions of FAI induce variable damage to the hip joint, with cam lesions preferentially affecting the acetabular cartilage and pincer lesions affecting the labrum and peripheral acetabulum in a more circumferential manner (16).

## Who Gets FAI?

The collective understanding of the etiology, history, and clinical presentation of FAI has evolved dramatically over the past decade. As FAI represents a syndrome with varying degrees of bony, chondral, and labral pathology at the hip joint, its presentation is similarly diverse. FAI is frequently seen in athletes. One recent systematic review of North American patients undergoing surgery for FAI found that the average age at surgery was 28 years and there was a mild female preponderance FAI at 55% of patients (9). Pincer FAI typically presents in middle-aged women; however, pincer lesions occur commonly in males as well (3, 9, 17). Cam lesions, on the other hand, demonstrate a near 3:1 male predominance and are seen more often in the younger population (3, 17). FAI can be present in the acute or chronic setting, and can be associated with prior trauma, such as malunion of a femoral neck fracture. It has also been associated with pediatric hip diseases, such as developmental dysplasia of the hip (DDH), slipped capital femoral epiphysis (SCFE), and Legg–Calve–Perthes disease (LCPD) (18, 19). Despite this, the most common presentation for FAI is idiopathic, atraumatic pain that has been ongoing between 12 and 16 months (9).

The precise etiology of FAI is still unclear; however, several theories exist linking genetic predisposition, pediatric deformity, and trauma, as well as high intensity adolescent athletic activity to the onset of FAI. Genetic factors involved in FAI pathogenesis were proposed by Pollard et al. who reported that siblings of patients with symptomatic FAI possessed an increased predilection for radiographic and clinical impingement signs (20). These findings, coupled with the increased incidence of cam FAI in males, promote the conclusion that there are intrinsic, although as of yet unidentified, genetic factors influencing hip morphology in the development of FAI (19, 21).

Additionally, pediatric hip disorders can predispose to FAI. SCFE deformities have been shown to predispose to the development of cam impingement in adulthood, which is mechanically consistent with the anterosuperior displacement of the femoral metaphysis in the pediatric disease (5, 19, 22, 23). Similarly, the natural history of LCPD can lead to FAI, in this case resulting from aspherical enlargement of the femoral head (coxa magna) representing the healed osteonecrotic epiphysis (23–25). Unlike SCFE, however, LCPD promotes both intra- and extra-articular impingement, complicating the nature of pain generation (19, 26, 27). Cam lesions have been found in the patients with prior femoral neck fractures, with Mathew et al. finding radiographic FAI in 84% of this cohort (28, 29). Furthermore, FAI can arise as a postsurgical consequence of the Bernese Peri-Acetabular Osteotomy, as the procedure can induce an iatrogenic pincer type acetabular conformation (30, 31). In general, any condition or procedure that alters the native bony anatomy of the hip joint can lead to clinical and radiological signs of impingement and secondary FAI.

While FAI is associated with prior hip pathology, it is most often idiopathic, and particularly common in the athletic population (3). This finding has led to multiple efforts investigating the relationship between sports participation and FAI development (19, 30, 32–37). One recent systematic review of 208 competitive male athletes (300 hips) concluded that athletes participating in high-impact sports (basketball, hockey, and soccer) were significantly more likely to develop cam lesions than non-athletes (odds ratio 1.9–8.0) (35). Furthermore, it has been proposed that the cam lesions develop in response to high intensity activity during development (5, 32, 38). In a study of 77 elite adolescent hockey players, Siebenrock et al. report higher alpha angles in athletes with closed physes as well as higher alpha angles in athletes reporting hip pain (39). In a recent prospective study of pre-professional adolescent soccer players, Agricola et al. measured proximal femur morphology at baseline and 2 years, finding significantly increased radiologic evidence of cam lesions at the 2-year time point (40). While there is evidence that suggests cam lesions can develop in high-intensity adolescent athletes, these studies primarily investigated a western European population. The prevalence of cam deformities in East Asian populations, however, is markedly reduced (41, 42). Thus, the role of genetics likely predisposes certain populations to FAI deformity under given repetitive and supra-physiologic loading conditions (21).

## Does FAI Predispose to Arthritis?

Based on multiple *in situ* observations of the impingement and damage patterns associated with FAI through open surgical dislocation of the femoral head, Ganz et al. proposed FAI as a precursor to the development of OA (1, 2). Their group highlighted the specific labral and chondral injuries affiliated with cam and pincer lesions and argued that prolonged contact between the deformed acetabulum and proximal femur promote further cartilage damage and eventual joint deterioration. Cam lesions, in particular, have demonstrated an increased risk for the development of OA (16, 43–46). One retrospective study analyzed the radiographs of patients with unilateral hip OA and found that the presence of a non-spherical femoral head as seen in cam lesions has a significant association with OA (45). Furthermore, one prospective study of Dutch patients demonstrated that moderate (alpha angle >60°) and severe cam deformities (alpha angle >83°) demonstrated a respective 3.7 and 10 times greater likelihood of developing OA over a 5-year time span when compared to controls (47). This study also identified a positive predictive value of 53% for the future development of OA in patients with cam deformities on X-ray and a positive impingement sign (47). Similarly, in a study investigating the prevalence of FAI deformities in patients undergoing THA for OA, found patients younger than 65 undergoing THA were more likely to have evidence of cam, but not pincer lesions (48). Thus, while cam lesions are linked to the development of OA, the relevance of pincer lesions in OA are less clear (44, 49). However, as isolated pincer lesions are rare, seen in only 7% of FAI cases, the cam lesion present in the other 93% of FAI cases may be the primary driver of OA in FAI (9).

In addition to the epidemiologic and radiographic studies correlating OA development to characteristic FAI lesions, biomarkers seen in OA are being investigated to identify correlations to patients with FAI [Table 2 (72–80)]. While there are over 70 biomarkers that have been studied in OA, validation has proved challenging (50). One recent systematic review identified six biomarkers that were correlated to OA progression: cartilage oligomeric protein (COMP), 25-OH vitamin D, N-terminal telopeptide (NTX), type II collagen C telopeptide (CTX-II), TIMP metalloproteinase inhibitor (TIMP), and vascular cell adhesion molecule 1 (VCAM-1) (50). In support of COMP as a biomarker, Dragomir et al. found that COMP levels were higher in patients with clinical signs of hip dysfunction and Bedi et al. reported COMP levels to be significantly increased in male athletes with FAI compared to a control group (50–52). However, the relevance of COMP has been questioned by several studies that have not found associations between COMP and hip OA (50, 53, 54). One study suggests that deamidated COMP (DCOMP) may be a more useful biomarker as they found a strong association with DCOMP levels and radiographic OA, as well as higher DCOMP concentrations in regions in proximity with OA lesions (54). Additionally, Bedi et al. also found a 276% increase in circulating CRP levels in patients with FAI compared to controls, indicating that there may be an inflammatory component to FAI (52). This observation was supported by a recent histologic study which found significantly increased macrophage and mast cell expression in labrums from patients with FAI compared to labrums from patients with OA (55). While there are currently no validated biomarkers for FAI, studies have shown promising associations that must be confirmed by future research (50).

**Table 2:** **Molecular biomarkers associated with the onset and/or progression of hip osteoarthritis and their relation to FAI**.

Biomarker	Relation to hip OA	Relation to FAI
Cartilage oligomeric protein (COMP) (52, 74)	Higher levels may be associated with hip OA progression	Elevated in male athletes with FAI (1 study)
25-OH vitamin D (75)	Lower levels may be associated with worsening Hip OA	
N-terminal telopeptide (NTX) (76)	Higher levels may be associated with hip OA progression	
Urine type II collagen C telopeptide (CTX-II) (77)	Higher levels associated with hip OA progression	
Tissue inhibitor of metalloproteinase-1 (TIMP) (78)	Higher levels may be associated with hip OA progression	
Vascular cell adhesion molecule 1 (VCAM-1) (79)	Higher levels may be associated with hip OA progression	
C-reactive protein (CRP) (52)		Higher in FAI patients compared to non-FAI patients (1 study)
Synovial fibronectin-aggrecan complex(sf-FAC) (80)		Higher in patients undergoing surgery for hip replacement compared to hip arthroscopy (1 study)

While cam or pincer deformities are a necessary condition for FAI, they are not pathognomonic and are frequently encountered in the asymptomatic population. One recent systematic review of 26 studies with 2114 total asymptomatic hips found an average cam and pincer lesion prevalence of 37 and 63%, respectively (4). Previously reported percentages of cam lesions ranged between 10 and 24%, and the authors attribute their reported increase to the high percentage of athletes in the review population (56–58). Cam lesions, measured on MRI as well as AP and modified Dunn radiographs, are variably defined by alpha angle and standardized cutoff values for normal and abnormal alpha angles are lacking (59–61). Pincer lesion prevalence may also be over-reported as radiographic findings, such as the cross over and posterior wall signs have proven unreliable markers (4, 62). Despite this, it is clear that the radiographic findings of FAI are common in the asymptomatic population, which has brought the correlation between cam and pincer deformities and OA into question (63). One putative explanation for this discrepancy lies in status patient’s articular cartilage. Hogervorst et al. introduced the term “cartilotype” to assess the susceptibility of cartilage to degradation in response to mechanical stress (21). Thus, patients with radiographic FAI may remain asymptomatic if their articular cartilage is able to withstand the impingement produced by the osseous deformities (64). Taken together, the surgeon should relate the patient’s clinical history and findings on physical exam to the radiographic evidence when preparing for the surgical correction of FAI.

## Does Surgical Treatment Alter the Natural History?

A number of studies have demonstrated that surgery for FAI is a safe and effective means to improve function and decrease pain levels in the short- and mid-term (65–67). Generally, open and arthroscopic treatment modalities appear to provide comparable outcomes in the mid-term aside from general health-related quality of life, which is significantly higher in the arthroscopic group (68). Intuitively, it makes sense that surgical intervention to remove the osseous mechanical blocks to motion will prevent further damage to the soft tissue structures (cartilage and labrum) of the hip. Studies have corroborated that the severity of cartilaginous and labral degenerative changes are directly associated with the duration of the underlying pathology (69–71). However, the available literature to date cannot assure that surgical intervention either in the asymptomatic or symptomatic patient will prevent the progression to OA and the risk of eventually requiring a THA. Prognostic indicators of early OA following treatment of FAI have yet to be elucidated, thus it is difficult at this time to associate treatment of FAI and the progression of OA.

Research strategies to further investigate the natural history of FAI and the association with OA are currently underway. Such efforts focus on prospective evaluations of younger patients with an early diagnosis of FAI. This study design enables researchers to focus on early interventions that may change the disease course over a long period of time. This study design, ideally in a randomized fashion, will aid in answering long-term questions regarding surgical intervention (both arthroscopic and open) and the ability of these interventions to delay or prevent OA and the need for THA.

## Conclusion

The purpose of this article is to review our current understanding of FAI by focusing on the natural history of the disease process. Surgical correction of the underlying osseous pathology in the symptomatic patient will improve function and decrease pain. Although an association between FAI and the development of OA is logical, long-term longitudinal studies have not yet been completed to substantiate cause and effect. Therefore, there currently is insufficient evidence to recommend prophylactic surgery in asymptomatic patients with radiographic evidence of FAI. Future studies targeting the early diagnosis and treatment of FAI will assist in elucidating the etiology of FAI, the natural history of the disease process, and ultimately the association between FAI and the progression to hip OA.

## Conflict of Interest Statement

Thomas H. Wuerz MD: Paid consultant, CONMED Linvatec. The remaining co-authors declare that the research was conducted in the absence of any commercial or financial relationships that could be construed as a potential conflict of interest.
